# Hdac3 deletion in myeloid progenitor cells enhances bone healing in females and limits osteoclast fusion via Pmepa1

**DOI:** 10.1038/s41598-020-78364-5

**Published:** 2020-12-11

**Authors:** David H. H. Molstad, Elizabeth Zars, Andrew Norton, Kim C. Mansky, Jennifer J. Westendorf, Elizabeth W. Bradley

**Affiliations:** 1 Department of Orthopedics, University of Minnesota, Elizabeth W. Bradley, 100 Church St. S.E., Minneapolis, MN 55455 USA; 2 Stem Cell Institute, University of Minnesota, Minneapolis, MN USA; 3 Developmental and Surgical Sciences, University of Minnesota, Minneapolis, MN USA; 4 Departments of Orthopedic Surgery, Mayo Clinic, Rochester, MN USA; 5 Biochemistry and Molecular Biology, Mayo Clinic, Rochester, MN USA

**Keywords:** Cell biology, Diseases, Medical research

## Abstract

Previous studies examining the role of the histone deacetylase Hdac3 within myeloid cells demonstrated that Hdac3 promotes M2 activation and tissue healing in inflammatory conditions. Since myeloid lineage cells are required for proper bone formation and regeneration, in this study we examined the functions of Hdac3 during bone healing. Conditional deletion of Hdac3 within myeloid progenitors accelerates healing of cortical bone defects. Moreover, reduced osteoclast numbers within the defect site are correlated with Hdac3 suppression. Ex vivo osteoclastogenesis assays further demonstrate that Hdac3 deficiency limits osteoclastogenesis, the number of nuclei per cell and bone resorption, suggesting a defect in cell fusion. High throughput RNA sequencing identified the transmembrane protein Pmepa1 as a differentially expressed gene within osteoclast progenitor cells. Knockdown of Pmepa1 partially restores defects in osteoclastogenesis induced by Hdac3 deficiency. These results show that Hdac3 is required for optimal bone healing and osteoclast fusion, potentially via its regulation of Pmepa1 expression.

## Introduction

An estimated 5–10% of bone fractures will exhibit healing complications including delayed healing or non-union^1,2^. While many factors can limit healing, enhanced inflammation and altered immune responses are significant contributors^3,4^. Macrophages participate in inflammation and are recruited to sites of infection and damage where they play a central role in the tissue regeneration process. Macrophages contribute to both the initial injury response as well as to subsequent tissue healing. Although a spectrum of polarization exists, macrophages can be classified into two primary polarization states. Inflammatory macrophages (e.g., M1) are primarily involved during the inflammatory phase, whereas alternative macrophages (e.g., M2) are effectors of the tissue healing phase^5^. M1 respond to inflammatory mediators such as IFNγ, LPS and TNFα, but M2 are stimulated by IL4 and IL13^6^. These immune cells contribute to bone healing following injury^7–10^. Both circulating macrophages and tissue resident macrophages within bone (e.g., osteomacs) contribute to bone healing^11,12^, and osteomacs aid in bone growth during development and promote bone formation^8,9,11,13–22^.

Osteoclasts are large, multinucleated cells that resorb bone. They arise from the fusion of myeloid progenitor cells, a process which is facilitated by two cytokines, M-CSF and RANKL. Although polarized macrophages do not give rise to osteoclasts following in vitro exposure to RANKL^23,24^, polarized macrophages can influence the differentiation osteoclast lineage cells. M2 are reported to suppress osteoclastogenesis via production of IL4 and IL10 and consequent repression of NFATc1^25–30^. Conversely, inflammatory mediators produced by M1, including IFNγ, TNFα and IL6, promote either the proliferation of pre-osteoclast cells or their differentiation to the osteoclast lineage^31–38^. As these cells aid in healing and repair, macrophage polarization state may influence bone healing responses.

Histone deacetylases (Hdacs) are enzymes that remove acetyl groups from lysine side chains of histones and other proteins. Hdacs regulate numerous cellular and mitochondrial processes including gene transcription, DNA repair, protein stability, cytoskeletal dynamics, and signaling pathways to affect both development and aging^39^. Hdac3 is expressed by a wide variety of cell types and is predominantly localized to the nucleus. Germline deletion of Hdac3 results in embryonic lethality^40^, but conditional deletion revealed a central role for Hdac3 in facilitating endochondral and intramembranous ossification and promoting long bone growth^41,42^. Mesenchymal lineage Hdac3 is essential to maintain bone mass during aging and functions to suppress bone marrow adiposity^43–46^. Conditional deletion of Hdac3 within the monocyte/macrophage lineage (LysM-Cre) promotes alternative macrophage marker gene expression and hyper-responsiveness to IL4^47^. Moreover, Hdac3 induces half of the inflammatory gene program of macrophages following stimulation with LPS, and Hdac3 deficiency enhances collagen deposition within atherosclerotic lesions and stabilizes plaques^48,49^. Importantly, Hdac inhibitors act as anti-inflammatory agents in mice and humans^50–52^; thus, Hdac3 inhibition may temper inflammatory responses of M1 within the periphery. Given the importance of macrophages during tissue healing, we examined the requirement of myeloid lineage-specific Hdac3 to bone healing and osteoclastogenesis. We find that 12-week-old female Hdac3-cKO_LysM_ mice demonstrate more bone volume within cortical bone defects and limited osteoclast fusion.

## Results

### Deletion of Hdac3 in macrophage lineage cells confers alternate macrophage activation

**Figure 1:**
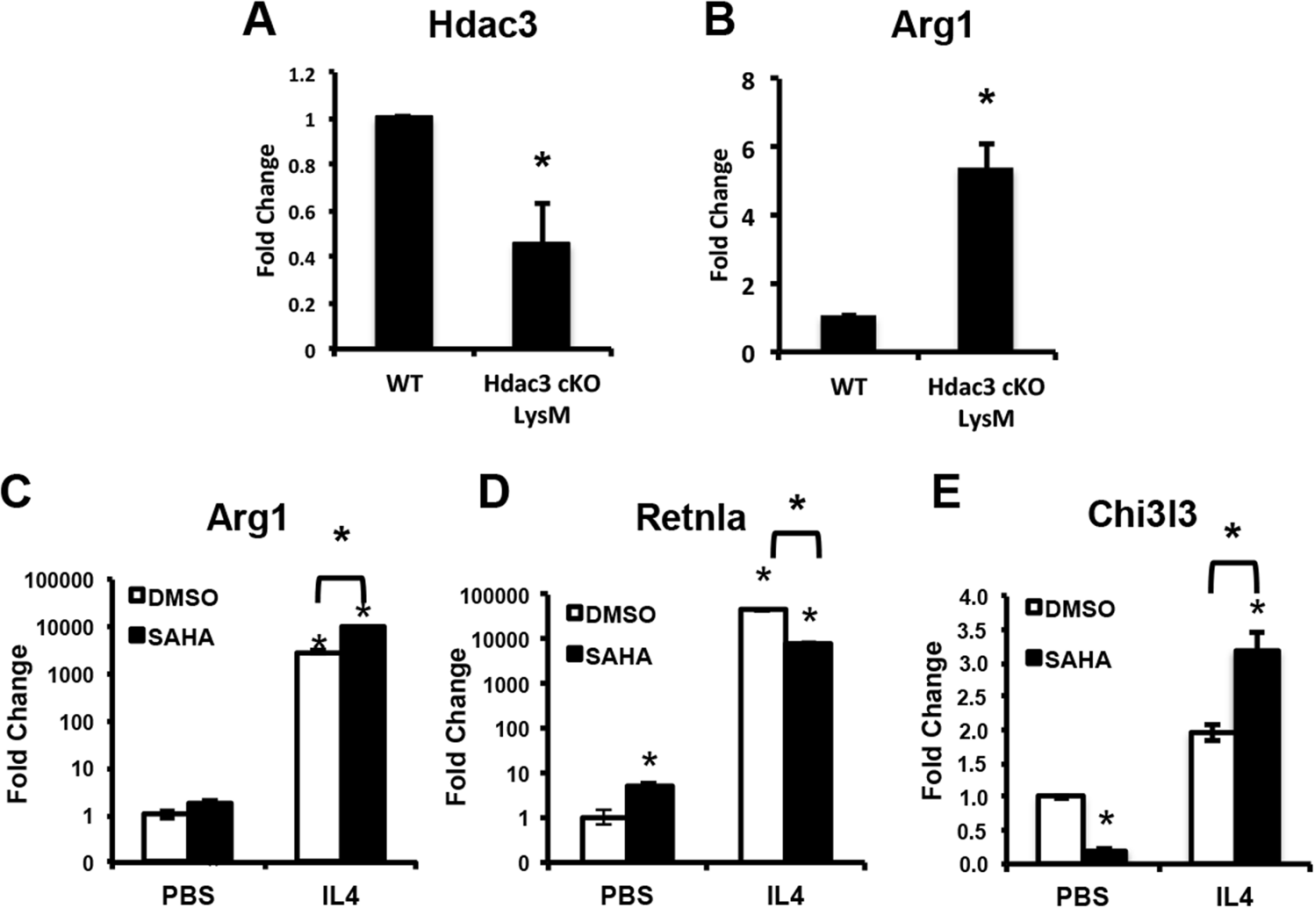
Hdac3 deficiency or Hdac inhibition promotes expression of alternative macrophage activation markers. (**A**–**D**) Bone marrow macrophages were isolated and cultured for 24 h. Expression of Hdac3 (**A**) and Arg1 (**B**) was evaluated by qPCR. *p < 0.05. (**C**–**E**) Bone marrow macrophages were isolated in the presence of the Hdac inhibitor SAHA or vehicle control. Cells were then exposed to IL4 for 24 h in the presence of SAHA. Expression of Arg1 (**C**), Retnla (**D**) and Chi313 (**E**) was evaluated by qPCR. *p < 0.05.

Prior work demonstrated that conditional deletion of Hdac3 within bone marrow cells expressing LysM-Cre enhanced alternative macrophage activation^47^. We isolated bone marrow macrophages from male or female Hdac3 cKO_LysM_ mice or their sex-matched WT control Cre^+^ littermates and confirmed elevated levels of the alternative macrophage marker Arg1 (Fig. 1A,B). BMMs exposed to the pan Hdac inhibitor SAHA for 24 h also exhibited increased expression of alternative macrophage activation markers and hyper-responsiveness to IL4 (Fig. 1C–E), known hallmarks of alternative macrophage activation. These data confirm prior observations made by Mullican et al.^47^.

### Conditional deletion of Hdac3 enhances cortical bone healing

**Figure 2:**
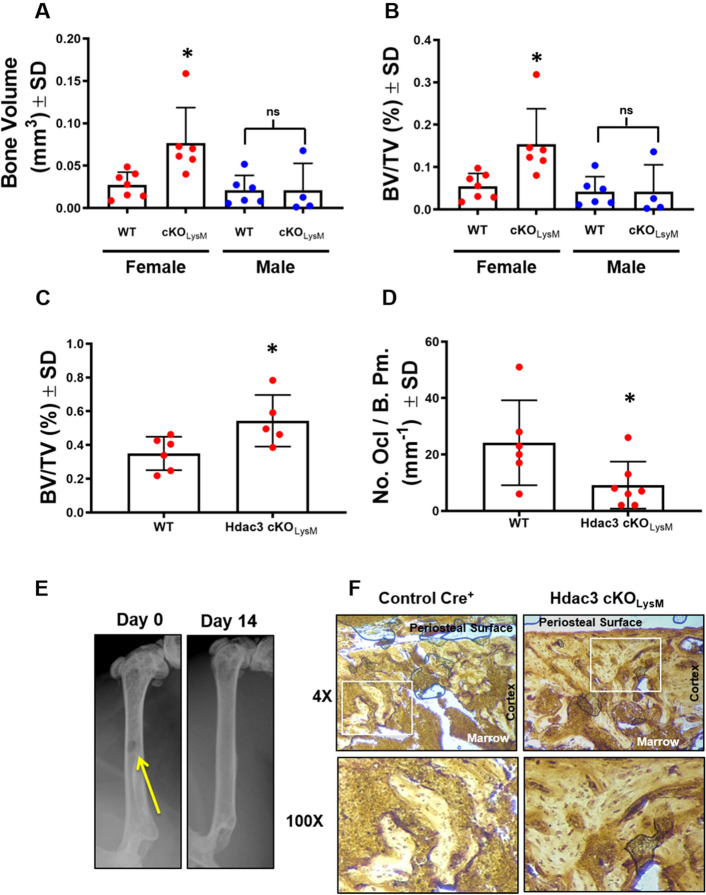
Monocyte/Macrophage lineage Hdac3 deficiency enhances bone healing. (**A**–**D**) Hdac3 cKO_LysM_ mice and their Control Cre^+^ littermates were aged to 12 weeks. Single cortex bone defects were generated in the left femur and bone healing was assessed after 14 days. Micro computed-tomography was performed by blinded study staff and used to determine (**A**) bone volume and (**B**) BV/TV within the defect. Femurs were sectioned and stained with Goldner’s trichrome. Blinded study staff determined (**C**) BV/TV or (**D**) TRAP to define the number of osteoclasts within the defect site. *p < 0.05 (**E**) Radiographs showing cortical bone defects at day 0 and day 14. (**F**) TRAP staining at the defect site.

**Figure 3:**
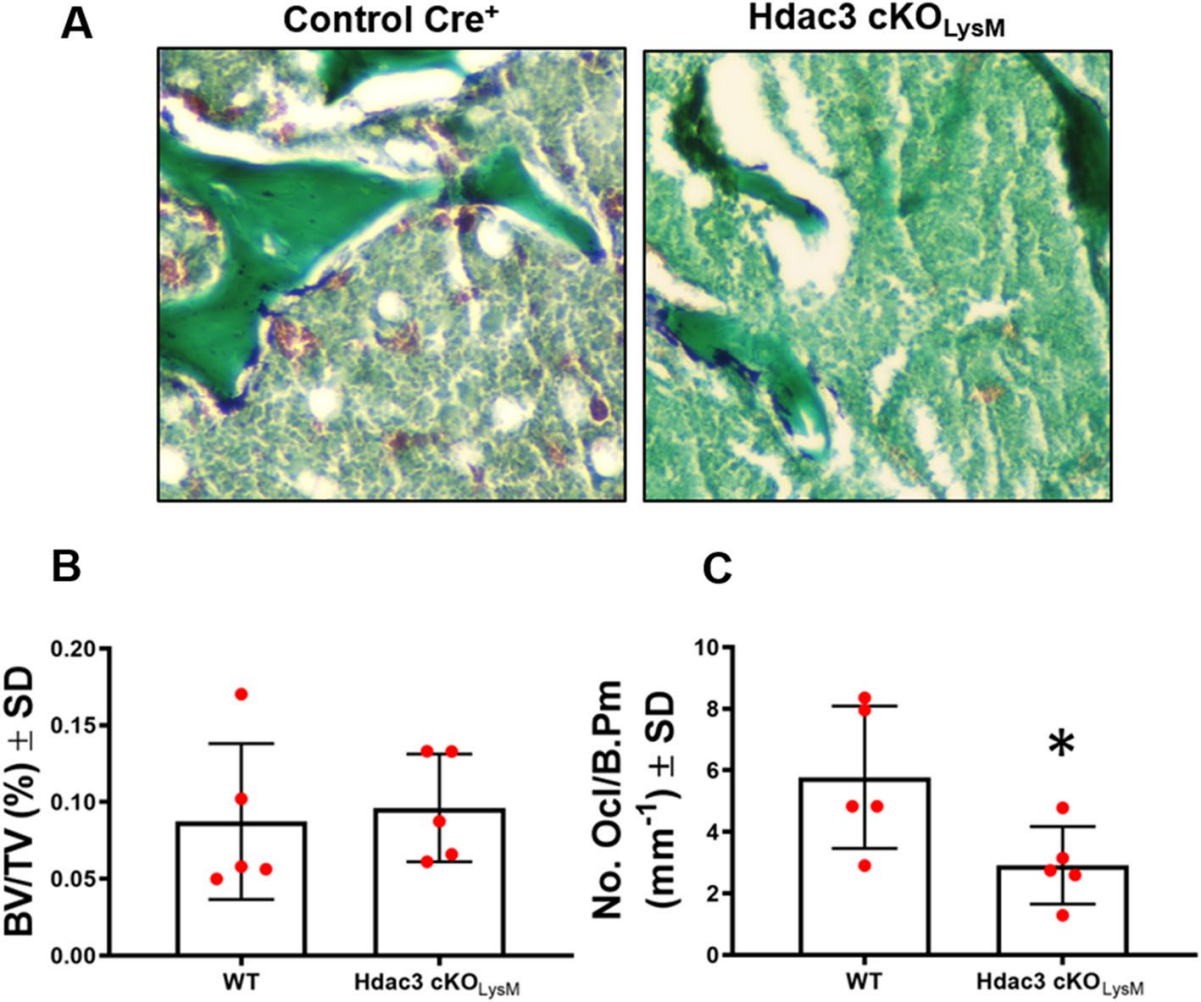
Hdac3 suppression limits osteoclast numbers. Hdac3 cKO_LysM_ mice and their Control Cre^+^ littermates were aged to 12 weeks. Femurs were sectioned and stained for TRAP and counterstained with fast green (**A**) and blinded study staff determined BV/TV (**B**) and defined the number of osteoclasts (**C**) within the distal femur. *p < 0.05.

Macrophage activation can influence how tissues heal and regenerate. Hdac3-deficiency within the macrophage lineage limits atherosclerotic plaque lesions, but has no effect on spinal cord injury healing^49,53^. To determine if macrophage lineage Hdac3 expression affected bone healing, we induced single cortex defects in male and female Hdac3 cKO_LysM_ 12-week-old mice or their Cre^+^ control littermates and assessed bone healing 14 days after defect generation. Radiography confirmed generation and uniform size of each induced defect (Fig. 2E). Blinded analyses revealed that Hdac3 cKO_LysM_ female mice showed an average 140% increase in bone volume and BV/TV within the defect site, but no changes were observed in males (Fig. 2A,B). Increased BV/TV within the defect site of females was confirmed via histomorphometry, with a greater amount of bone volume along the periosteal surface within the defect of Hdac3 cKO_LysM_ mice (Fig. 2C). More bone volume within the defect was accompanied by a significant reduction in osteoclasts per defect volume (Fig. 2D,E,F), but no change in osteoblast number was observed (data not shown). We also assessed the baseline cortical and trabecular parameters of the distal femur of intact 12-week-old male and female Hdac3 cKO_LysM_ mice compared to their control Cre^+^ littermates, but did not find a change in these parameters at this age (Table 1). We confirmed these results via histomorphometry, but observed diminished osteoclast numbers within the distal femur (Fig. 3A–C).

**Table 1:** Distal femur bone parameters of intact 12-week-old Hdac3 cKO_LysM_ mice assessed by µCT.

	BV/TV	Conn D	SMI	Tb. N	Tb. Th	Tb. Sp	Cort Th	Cort BMD
Female								
WT (n = 10)	**0.039 ± 0.02**	**12.02 ± 14**	**2.91 ± 0.37**	**1.86 ± 0.57**	**0.041 ± 0.005**	**0.39 ± 0.11**	**0.15 ± 0.01**	**400.265 ± 19**
Hdac3 cKO_LysM_ (n = 5)	**0.036 ± 0.01**	**13.3 ± 2.3**	**2.86 ± 0.14**	**1.84 ± 0.52**	**0.040 ± 0.004**	**0.40 ± 0.14**	**0.14 ± 0.01**	**387.003 ± 8**
*p* value	**0.803**	**0.888**	**0.841**	**0.940**	**0.665**	**0.797**	**0.551**	**0.589**
Male								
WT (n = 9)	**0.094 ± 0.04**	**61.81 ± 36**	**2.71 ± 0.35**	**3.17 ± 1.02**	**0.042 ± 0.007**	**0.23 ± 0.04**	**0.14 ± 0.03**	**410.4 ± 47**
Hdac3 cKO_LysM_ (n = 7)	**0.093 ± 0.04**	**79.36 ± 42**	**2.56 ± 0.27**	**3.57 ± 1.04**	**0.044 ± 0.006**	**0.23 ± 0.05**	**0.15 ± 0.03**	**364.4 ± 43**
*p* value	**0.961**	**0.525**	**0.493**	**0.458**	**0.661**	**0.937**	**0.632**	**0.167**

### Hdac3 is required for optimal osteoclast fusion

**Figure 4:**
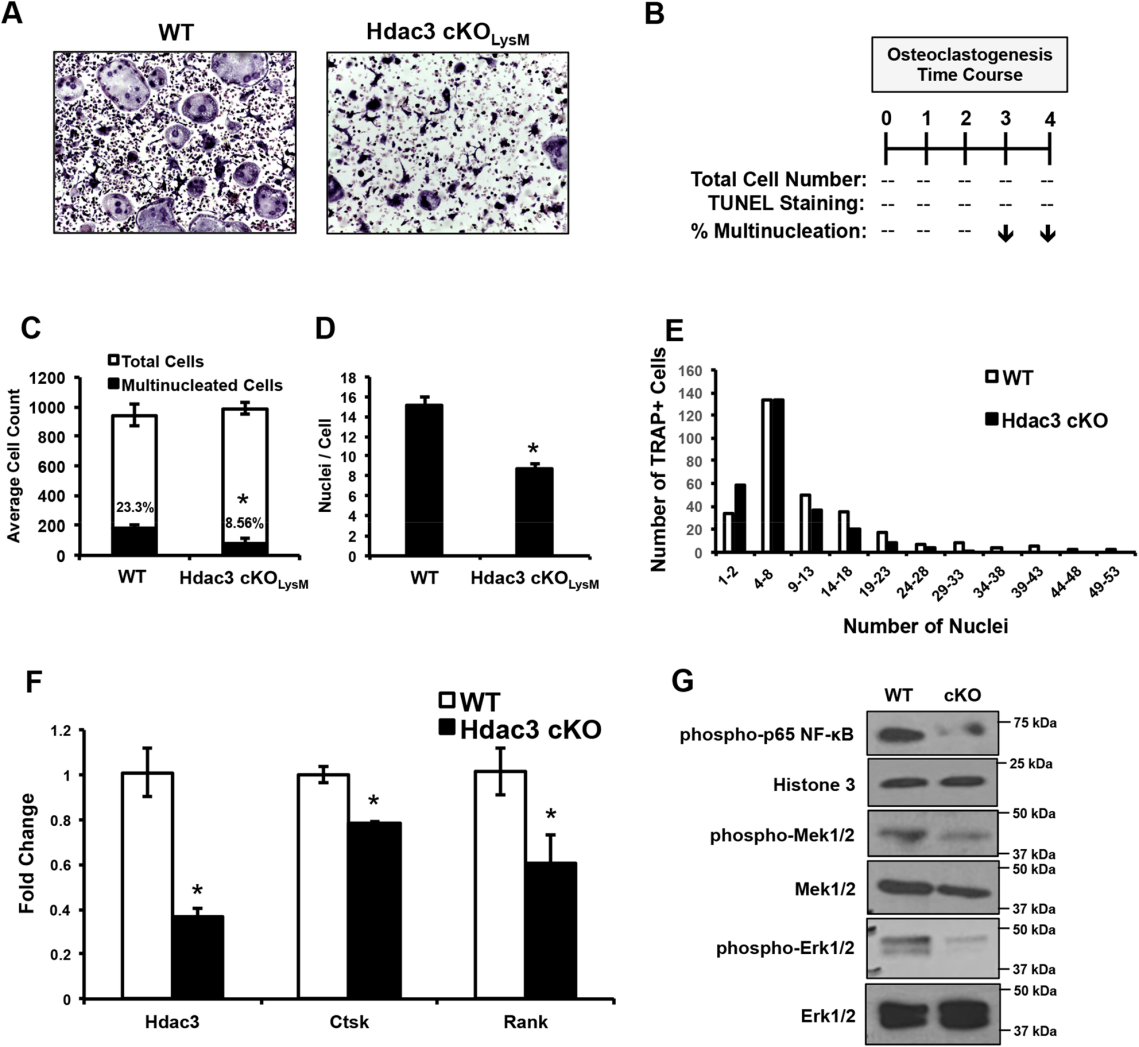
Monocyte/Macrophage lineage Hdac3 deficiency decreases ex vivo osteoclastogenesis. Bone marrow macrophages were collected from Hdac3 cKO_LysM_ 6-week-old male or female mice or their control Cre^+^ littermates and used to generate osteoclasts ex vivo. Shown are cultures from female mice. TRAP staining was performed (**A**). An osteoclastogenesis time course was performed (**B**) and the percent multi-nucleated cells (**C**) number of nuclei per cell (**D**) and occurrence of cells with a defined number of nuclei was determined (**E**). *p < 0.05. (**F**) Hdac3 levels and those of early osteoclastogenic genes Ctsk and RANK were assessed by qPCR. *p < 0.05. (**G**) Western blotting was performed as shown.

We observed decreased osteoclast numbers within female Hdac3 cKO_LysM_ mice in vivo and Hdac3 knockdown in bone marrow macrophages was shown to reduce osteoclastogenesis^54^, but the mechanism behind this observation was not explored. To address the effects of Hdac3 conditional deletion on commitment of progenitor cells to the osteoclast lineage, we performed ex vivo osteoclastogenesis assays. Deletion of Hdac3 resulted in decreased numbers of multinucleated osteoclasts on day 4 (Fig. 4A). We performed an osteoclastogenesis time course and found that while the number of mono- or multinucleated cells was not altered early, at the onset of osteoclast fusion (day 3) the percentage of Hdac3 cKO_LysM_ multinucleated cells was suppressed by 14.7 percent (Fig. 4B,C), whereas no change in the total number or TUNEL positive cells was observed (data not shown). This suggested a potential defect in osteoclast fusion. We therefore determined the number of nuclei per cell and found that osteoclasts derived from Hdac3 cKO_LysM_ mice had reduced numbers of nuclei per multinucleated cell (Fig. 4D). We also delineated the number of cells with a defined number of nuclei. Hdac3 deficiency enhanced the number of mononuclear cells (Fig. 4E). There was no difference in cells with 4–8 nuclei, but Hdac3 deficiency decreased the occurrence of cells with eight or more nuclei (Fig. 4E). Expression of early osteoclastogenic genes including Ctsk and RANK also decreased within Hdac3-depleted cells (Fig. 4F). Moreover, activation of signaling downstream of RANKL, including phosphorylation of p65 NF-κB and Mek/Erk, was diminished within Hdac3 cKO_LysM_ osteoclasts (Fig. 4G). These results demonstrate that Hdac3 deficiency suppresses osteoclast fusion.

To perform an unbiased assessment of the effects of Hdac3 deficiency during osteoclastogenesis, we performed high-throughput RNA sequencing. BMMs were collected from Hdac3 cKO_LysM_ 6-week-old male mice or their control littermates and cultured in osteoclastogenesis assays with RANKL and M-CSF. On day 3, when the defect in cell fusion was first observed, we collected mRNA for high-throughput sequencing. Using a false discovery rate of *p* < 0.05, we identified 71 differentially expressed genes versus sex-matched control littermates that had a fold change of two or greater (Table 2). Functional annotation using DAVID showed that the top categories affected by Hdac3 deficiency included Membrane, Extracellular Region and Cell Adhesion, all potentially involved in osteoclast progenitor fusion (Table 3).

**Table 2:** Genes differentially regulated by Hdac3 deficiency. RNA from day 3 Hdac3 cKO_LysM_ mice or wild type littermates was collected and used for high-throughput sequencing. Listed genes represent those with two-fold change or greater with an FDR < 0.05.

Up regulated genes			
Gene name	Fold change	*p* value	FDR
Cdcp1	11.20	0.0000	0.0000
Ccl7	6.34	0.0000	0.0000
Arg1	4.59	0.0000	0.0000
Ccl2	4.43	0.0000	0.0000
Serpinb9b	3.73	0.0000	0.0004
Rgs1	3.32	0.0000	0.0000
Cd276	3.23	0.0000	0.0006
Gdf3	2.77	0.0001	0.0104
Angptl2	2.75	0.0000	0.0002
Plau	2.67	0.0000	0.0002
Prss46	2.65	0.0001	0.0159
Cd36	2.63	0.0000	0.0000
Npy	2.56	0.0000	0.0007
Kitl	2.55	0.0000	0.0012
Gdf15	2.51	0.0000	0.0000
Flnc	2.51	0.0000	0.0042
Htra3	2.41	0.0002	0.0196
Efr3b	2.37	0.0001	0.0137
**Pmepa1**	2.23	0.0006	0.0457
Prdx1	2.15	0.0000	0.0000
Mt3	2.15	0.0004	0.0374
Pdpn	2.13	0.0001	0.0124
Dcstamp	2.10	0.0006	0.0465
**Down Regulated Genes**			
Sorbs3	0.26	0.0000	0.0061
Saa3	0.27	0.0000	0.0002
Fxyd2	0.27	0.0000	0.0003
Cd4	0.28	0.0000	0.0006
Lrg1	0.29	0.0000	0.0004
Nat8l	0.30	0.0000	0.0007
Kcnj10	0.30	0.0000	0.0051
Crispld2	0.31	0.0000	0.0049
Coro2b	0.32	0.0000	0.0004
Mapk8ip1	0.34	0.0000	0.0016
Camp	0.35	0.0000	0.0000
Ltf	0.35	0.0000	0.0000
Gpr34	0.35	0.0001	0.0115
Ngp	0.35	0.0000	0.0000
Emr4	0.36	0.0001	0.0163
Chil1	0.36	0.0000	0.0013
Slit1	0.37	0.0007	0.0490
Lcn2	0.37	0.0000	0.0000
Itgb2l	0.39	0.0000	0.0011
Mogat2	0.39	0.0003	0.0276
S100a9	0.40	0.0000	0.0000
Chil3	0.40	0.0000	0.0000
Fcrls	0.40	0.0000	0.0009
Abca9	0.41	0.0000	0.0033
Cyp27a1	0.41	0.0000	0.0043
Cd177	0.41	0.0000	0.0039
Nuak1	0.42	0.0003	0.0295
Gpx3	0.42	0.0000	0.0049
S100a8	0.42	0.0000	0.0000
Tmem176b	0.43	0.0000	0.0007
Itga9	0.43	0.0004	0.0375
Tmem176a	0.43	0.0000	0.0049
Pglyrp1	0.44	0.0000	0.0019
Abca13	0.44	0.0000	0.0006
Cd55	0.44	0.0000	0.0006
Slc9a7	0.44	0.0000	0.0039
P2ry14	0.44	0.0000	0.0000
Gpr162	0.45	0.0000	0.0025
Cd83	0.45	0.0000	0.0010
Slc46a3	0.45	0.0003	0.0284
Slc9a9	0.46	0.0003	0.0283
Abca6	0.47	0.0001	0.0142
Slc6a12	0.47	0.0000	0.0052
Fcnb	0.48	0.0000	0.0026
Gpr160	0.49	0.0001	0.0097
Esr1	0.50	0.0001	0.0099
Cfh	0.50	0.0001	0.0098

**Table 3:** Top functional categories affected by Hdac3 deficiency. Differentially regulated genes from high-throughput sequencing results were grouped into functional categories using the DAVID bioinformatics database.

Term	Count	*P* value	Benjamini
Cytoplasm	43	2.60E−02	5.40E−01
Membrane	43	6.00E−02	6.40E−01
Signal	42	3.50E−07	1.90E−05
Disulfide bond	39	3.40E−10	5.60E−08
Glycoprotein	36	2.90E−06	1.20E−04
Glycosylation site:N-linked (GlcNAc…)	33	4.20E−04	1.10E−01
Secreted	28	9.30E−10	7.80E−08
Extracellular region	28	4.50E−08	3.30E−06
Extracellular exosome	26	8.50E−04	4.10E−02
Hydrolase	12	8.80E−02	5.20E−01
Signal transduction	11	9.90E−02	8.00E−01
Immunity	10	5.20E−05	1.70E−03
Cell adhesion	8	1.10E−02	4.80E−01
Calcium	8	6.10E−02	4.60E−01
Negative regulation of transcription from RNA polymerase II promoter	8	7.40E−02	7.40E−01

**Figure 5:**
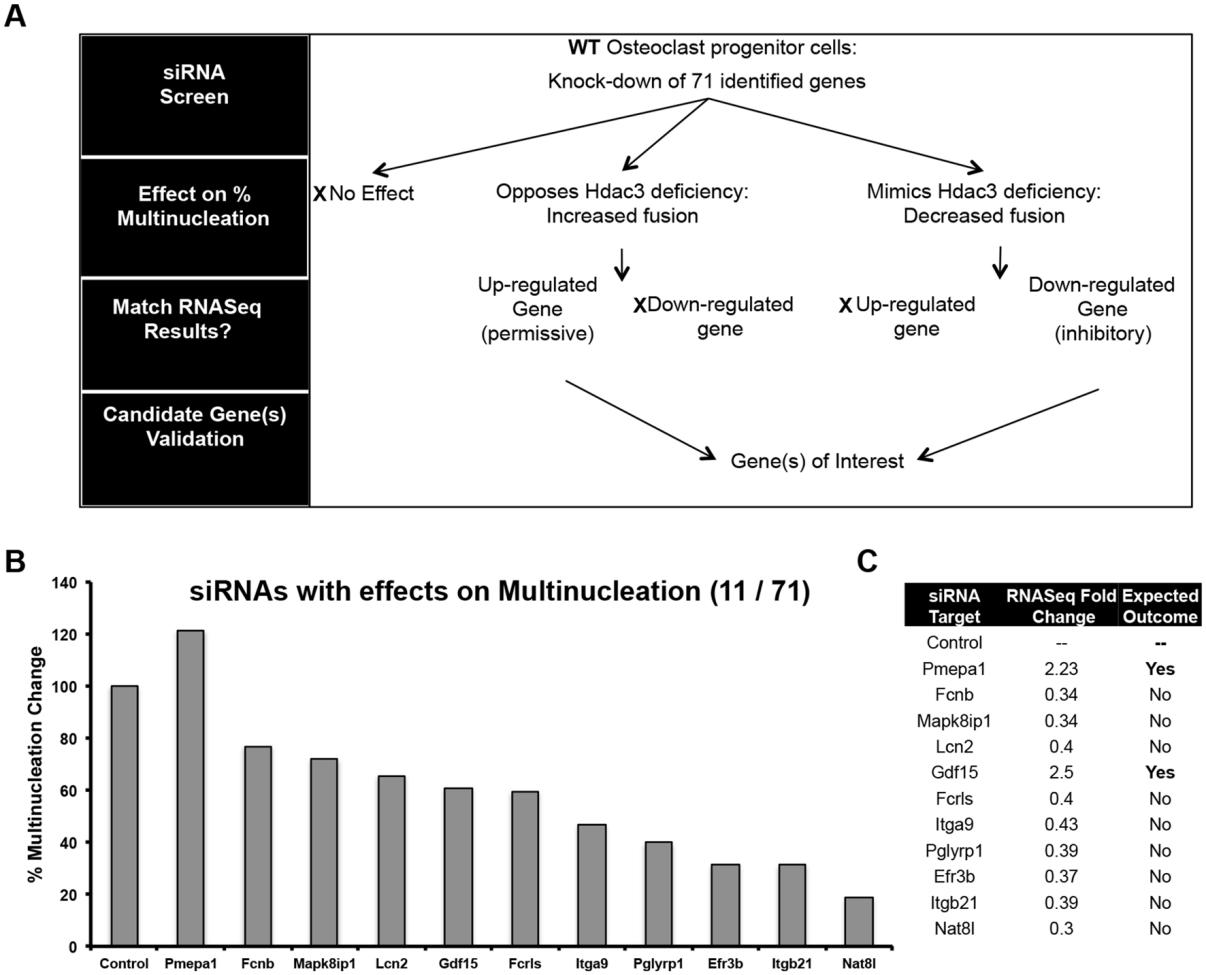
SiRNA-based screen to identify Hdac3 effector genes. A siRNA-based screen was performed as depicted in (A). Points where differentially expressed genes were excluded from the screen are noted with an X. Bone marrow macrophages were collected from wild type 6-week-old female mice. Cells were transfected with a siRNA targeting a differentially expressed gene or control siRNA on day 1 of osteoclast differentiation. Cells were then TRAP stained on day 4 and the targets that affected osteoclast multinucleation were identified (**B**). Genes that meet screening criteria outlined in (**A**) are highlighted (**C**).

We next knocked down each of the 71 differentially expressed genes in wildtype BMMs using a siRNA-based approach to identify Hdac3 effector genes involved in osteoclast fusion (Fig. 5). Wildtype BMMs were collected from male 6 to 8-week old mice. On day 1, cells were transfected with an siRNA smart pool targeting one of the 71 differentially expressed genes or a non-targeting control siRNA. On day 4, cultures were TRAP and DAPI stained and the number of mono- and multinucleated cells was determined. Using this approach, siRNAs that targeted genes up regulated by Hdac3 deficiency and increasing the percentage of multinucleated TRAP^+^ cells were considered positive; likewise, siRNAs targeting genes that were down regulated by Hdac3 deficiency that also resulted in reduced numbers of TRAP^+^ multinucleated cells were also considered (Fig. 5A). Knockdown of 11 out of 71 differentially expressed genes changed the percentage of multinucleated cells (Fig. 5B). Of these 11 genes, two (Pmepa1 and Gdf15) fit our criteria (Fig. 5C). Knockdown of Gdf15 did not rescue the effects of Hdac3 deficiency (data not shown).

### Pmepa1 knock-down rescues Hdac3-dependent defects in osteoclastogenesis

**Figure 6:**
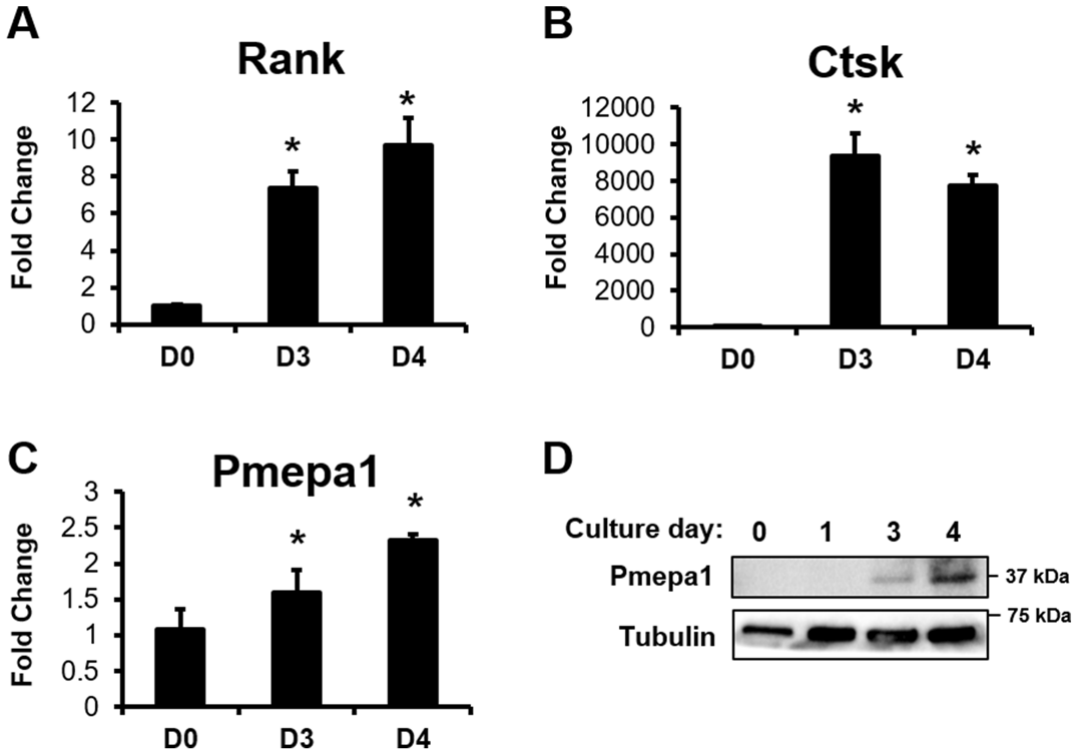
Pmepa1 expression increases during the fusion stage of osteoclastogenesis. Bone marrow macrophages were collected from WT 6-week-old male or female mice and cultured in the presence of RANKL and M-CSF for the indicated days. Shown are cultures from female mice. Expression levels of (**A**) Rank, (**B**) Ctsk and (**C**) Pmepa1 were evaluated by qPCR. *p < 0.05 (**D**) Western blotting was performed.

To further explore the role of Pmepa1 as a downstream target of Hdac3, we first assessed expression of Pmepa1 during different phases of osteoclast differentiation in WT cells. BMMs were isolated from 6 to 8 week-old WT mice and placed in ex vivo osteoclastogenesis assays for the indicated days (Fig. 6). Pmepa1 transcripts increased along with those of Ctsk and RANK (Fig. 6A–C). Protein levels of Pmepa1 were increased during phases of osteoclast fusion on days 3 and 4 (Fig. 6D).

**Figure 7:**
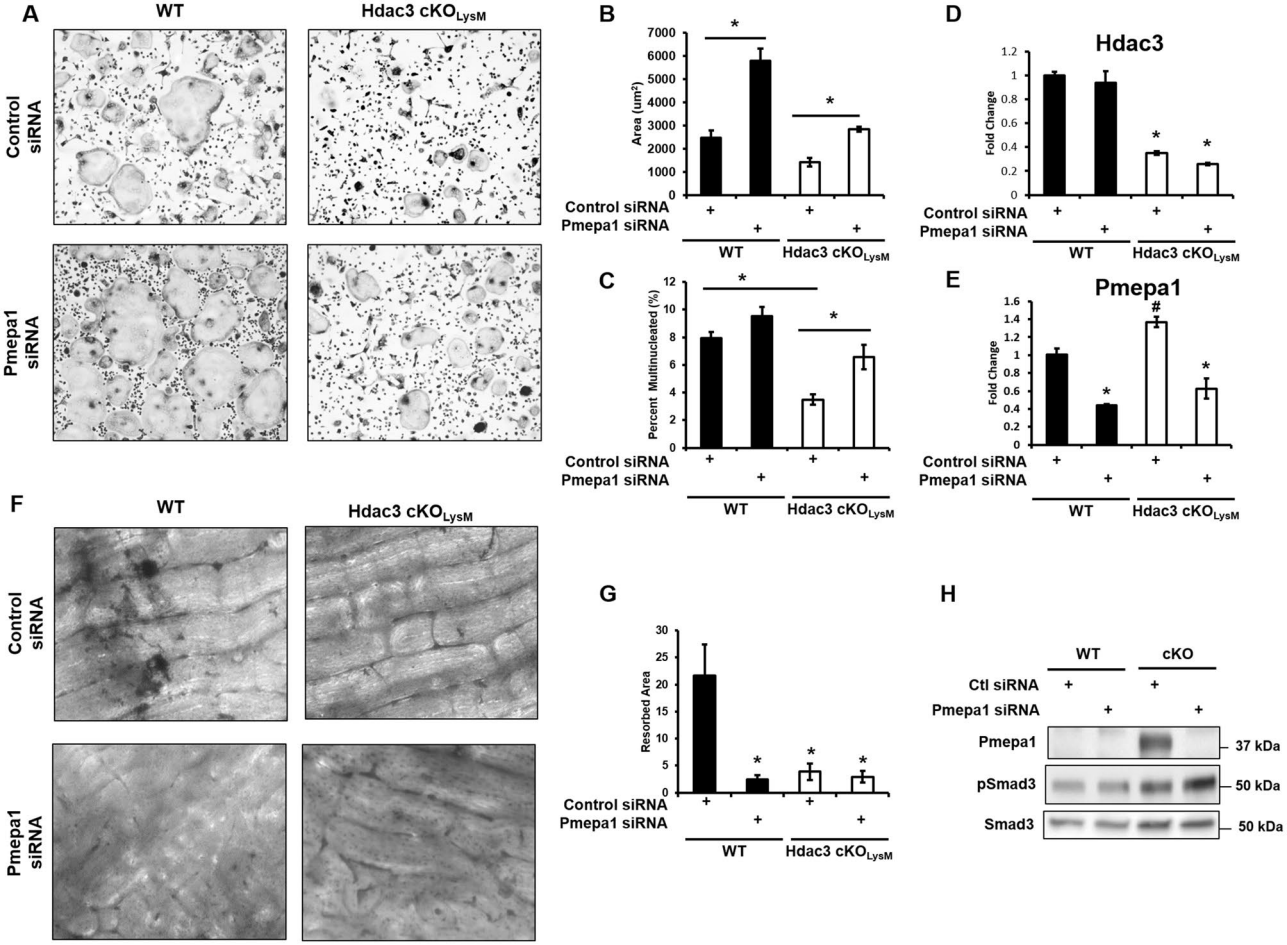
Pmepa1 knock-down rescues Hdac3 deficiency. Bone marrow macrophages were collected from Hdac3 cKO_LysM_ 6-week-old male or female mice or their control Cre^+^ littermates and used to generate osteoclasts ex vivo. Shown are cultures from female mice. On day 4 cells were TRAP stained (**A**) and (**B**) osteoclast area and (**C**) percent multinucleated cells was determined. *p < 0.05. Cells were also seeded onto bone slices. On day 14, resorption pits were stained with Toluidine blue (**F**) and quantified (**G**). *p < 0.05 Expression levels of (**D**) Hdac3 and (**E**) Pmepa1 were evaluated by qPCR. *p < 0.05 Western blotting confirmed knockdown of Pmepa1 (**F**). Pit formation assays were also performed (**H**).

BMMs were then isolated from 6–8 week-old male or female Hdac3 cKO_LysM_ mice and their sex-matched control littermates and placed in ex vivo culture with RANKL and M-CSF. On day 1, cells were transfected with siRNA smart pools targeting Pmepa1 or a non-targeting siRNA control. On day 4, cells were collected for analyses. Whereas deletion of Hdac3 decreased the number of multinucleated cells, knockdown of Pmepa1 in Hdac3 deficient cells significantly restored the size and number of multinucleated TRAP^+^ cells (Fig. 7A,B,C). Knockdown of Pmpea1 in control BMMs also enhanced the area and number of TRAP^+^ multinucleated cells (Fig. 7A,B,C). Real-time quantitative PCR verified suppression of Hdac3 levels in Hdac3 cKO_LysM_ cultures and knockdown of Pmepa1 (Fig. 7D,E). Western blotting also confirmed enhanced Pmepa1 levels in Hdac3 suppressed cells (Fig. 7H). Pmepa1 attenuates phosphorylation of Smad3; as expected, knockdown of Pmepa1 enhanced phospho-Smad3 levels (Fig. 7H). Bone marrow macrophages derived from Hdac3 cKO_LysM_ or their littermate controls were also seeded onto bone slices in osteoclastogenic conditions. On day 1, cells were transfected with Pmepa1 siRNAs or non-targeting siRNAs. Cultures were maintained for two weeks and the amount of resorbed bone was visualized. Less resorption occurred in cultures derived from Hdac3 cKO_LysM_ bone marrow macrophages, which was further enhanced by knockdown of Pmepa1 (Fig. 7F,G). Together these data demonstrate that Pmepa1 suppresses osteoclast fusion and is a downstream target of Hdac3-mediated repression.

## Discussion

Hdac inhibitors reduce inflammatory responses and alter macrophage polarization, but they also negatively impact the survival of highly proliferative cells such as mesenchymal progenitors^55–58^. In this study, we determined the effects of depleting Hdac3 within LysM-Cre-expressing cells on basal bone mass, cortical bone healing and osteoclastogenesis. We found that 12-week-old Hdac3 cKO_LysM_ mice had diminished osteoclast numbers, but no basal bone phenotype. In contrast, bone healing of Hdac3 cKO_LysM_ females was enhanced in a cortical bone injury model. Hdac3 is important for maintenance of bone mass during aging and its expression declines with age^57^. Although 12-week-old Hdac3 cKO_LysM_ do not have changes in basal bone mass, this does not preclude development of a phenotype with increased age. Deletion of Hdac3 enhanced cortical bone defect healing in female mice two weeks after injury, but not in male mice. This sex-specific effect may be due to loss of Hdac3/NCoR-dependent transcriptional repression of ERα target genes^59–62^. Since Hdac3 deficiency enhanced bone healing in a cortical defect model and Hdac inhibitors are protective against osteoarthritis^63^, future studies will be aimed at determining if deletion of Hdac3 within myeloid lineage cells likewise promotes healing of other skeletal tissues.

We also found that Hdac3 deficiency within myeloid progenitor cells diminished osteoclastogenesis, most likely due to decreased cell fusion. This is in line with previous reports demonstrating that knockdown of Hdac3 in bone marrow macrophages suppressed osteoclast numbers^54^. We used an siRNA-based screen to identify downstream targets of Hdac3 during osteoclastogenesis. This screen was performed using WT bone marrow macrophages and also identified other regulators of osteoclastogenesis; however, since we did not verify effective knock-down of all targets within this large siRNA-based screen, false-negative results are an inherent limitation. In general, Hdac3 functions to repress gene expression, but also has demonstrated coactivator functions^64^. For this reason we chose to target all genes that were differentially regulated by Hdac3 deficiency using an siRNA-based approach in WT bone marrow macrophages. Using this tactic, we identified Pmepa1, a transmembrane protein and inhibitor of Tgfβ signaling^65^.

Pmepa1 is induced by p38 MAPK signaling in response to RANKL, but expression is downregulated after 72 h in osteoclastogenesis assays^66^. Pmepa1 localizes to intracellular organelle membranes, and to cathepsin-K positive vesicle membranes in osteoclast progenitor cells^66^. Xu et al. showed that knockdown of Pmepa1 suppressed osteoclast activity and that Pmepa1 is associated with the actin ring of osteoclasts during adjuvant-induced bone resorption^67^. In our study, we find that knockdown of Pmepa1 after 24 h of RANKL exposure enhances osteoclast fusion, but represses osteoclast activity. In future studies, the mechanism by which Pmepa1 controls osteoclast fusion will be explored. Determining if suppression of Pmepa1 also aids in coupling to bone formation, similar to other models where osteoclastogenesis is enhanced but bone resorption is suppressed, is also of interest^68,69^.

Another limitation of this study is that we cannot separate the functions of Hdac3 in monocytes/macrophages from those required for osteoclastogenesis and osteoclast activity. Thus, the functions of Hdac3 within the committed osteoclasts will need to be explored with alternative Cre-drivers (e.g., TRAP-Cre, Ctsk-Cre) in future studies. Although our analyses suggests that osteoclast fusion is suppressed by Hdac3 deficiency, the exact mechanism by which Hdac3 limits bone healing is unclear in this model. Hdac3 depletion within the myeloid lineage may likewise have different effects on stages of bone healing (e.g., initial inflammatory response, later bone remodeling). Although osteoblast number was unaffected, this does not preclude an effect on osteoblast activity.

Together our data demonstrate that Hdac3 deficiency in LysM-Cre-expressing cells enhances bone healing and suppresses osteoclast fusion. Moreover, Pmepa1 may be a downstream target of Hdac3 controlling osteoclast fusion and activity.

## Methods

### Generation of Hdac3-LysM mice

Hdac3^fl/fl^ mice^40^ were crossed with mice expressing Cre-recombinase under the control of the LysM promoter^70^ to delete Hdac3 within LysM-expressing cells. Mice were genotyped for Cre as previously described^46^, or the Hdac3 floxed allele using the following primers: A: 5′-CCACTGGCTTCTCCTAAGTTC -3′, B: 5′- CCCAGGTTAGCTTTGAACTCT-3′ and C: 5′-TTTCCGTATTTGTGGAAGGA-3′. Conditional knockout animals from these crossings are referred to as Hdac3 cKO_LysM_ mice and are on the C57Bl/6 background. Cre^+^ control littermates from crossings were used as controls as appropriate. Animals were housed in an accredited facility under a 12-h light/dark cycle and provided water and food ad libitum. All animal research was conducted according to guidelines provided by the National Institute of Health and the Institute of Laboratory Animal Resources, National Research Council. The Mayo Clinic Institutional Animal Care and Use Committee approved all animal studies.

### Cortical bone defect generation

Single-cortex, fully stabilized defects were made in the mid-diaphysis of femurs as previously described^71^. Briefly, Cre^+^ male (n = 6) and female (n = 6) or Hdac3 cKO_LysM_ male (n = 5) and female (n = 7) mice were given 0.09 mg/kg buprenorphine perioperatively. Mice were then anesthetized with isoflurane and prepared for aseptic surgery. A small incision was made in the skin overlying the left hamstring and the femoral bone shaft was exposed by blunt dissection of the underlying muscle. A 0.7 mm diameter steel burr drill bit (#19007-07, Fine Science Tools, Foster City, CA) and an electric drill was used to induce a single-cortex defect in the mid-diaphysis of the femur. Defects were immediately irrigated with 1 ml sterile saline followed by incision closure. Defect healing was monitored via radiography 14 days after surgery. Mice were sacrificed by carbon dioxide inhalation at postoperative day 14.

### Micro-computed tomography

Bone architecture and mineralization were evaluated by ex vivo micro-CT. Femurs from 12-week-old male or female Hdac3 cKO_LysM_ mice (n = 7 males, n = 5 females) and their sex-matched Cre^+^ littermates (n = 9 males, n = 10 females) were isolated and fixed in 10% neutral buffered formalin for 24 h. Femurs were then stored in 70% ethanol prior to scanning at 70 kV, 221 ms with a 10.5 μm voxel size using a Scanco Viva40 micro-CT. For cortical bone analyses, a region of interest was defined at 10% of total femur length beginning at the femoral midpoint; defining the outer cortical shell and running a midshaft analysis with 260-threshold air filling correction analyzed samples. For trabecular measurements, a region of interest was defined at 10% of total femur length starting immediately proximal to the growth plate. Bone volume within cortical bone defects was performed as previously described^71^. Briefly, a region of interest at the defect site was identified and a defined total volume of 0.5 mm^3^ was assessed. Samples were analyzed using a 220-threshold air filling correction. Bone volume, BV/TV, bone mineral density, trabecular number (mm^−1^), trabecular thickness (mm), and trabecular separation (mm) were computed using the manufacturer’s software.

### Osteoclast cell culture, transfection, and treatment

Hind limbs were dissected from male or female 6 to 8-week-old WT, Cre^+^, Hdac3 cKO_LysM_ or Hdac3^fl/fl^ mice. Bone marrow macrophages were isolated as previously described^72^. Briefly, cells were flushed from the marrow cavity, pelleted and suspended in RBC Lysis Buffer (#00-4333-57, Invitrogen, Carlsbad, CA). Cells were pelleted and suspended in culture medium (phenol red-free alpha MEM, 10% FBS and 1% antibiotic/antimycotic) supplemented with 35 ng/mL M-CSF (#416-ML, R&D, Minneapolis, MN) and cultured overnight. Non-adherent cells were isolated and seeded at a density of 0.8 × 10^6^ cells/mL in 24-well plates (day 0) in culture medium supplemented with 35 ng/mL M-CSF, and 60 ng/mL RANKL (#315-11, Prepro Tech, Rocky Hill, NJ). Cultures were fed on day 3 with culture medium plus 35 ng/ml M-CSF, and 60 ng/mL RANKL. Cells were treated as described within the text and figures. For knockdown experiments, a custom siRNA ON-TARGET plus plate was designed (Dharmacon, Lafayette, CO) to screen differentially regulated genes of interest. SiRNA smart pools targeting Pmepa1 and Gdf15 (L-040445-00-0005 or L-043512-01-0005) or a control siRNA (D-001810-10-05) were also purchased from Dharmacon (Lafayette, CO,). Cells were transfected (day 1) with each siRNA using Lipofectamine RNAiMax at a 1:1 ratio (#13778075, Invitrogen) and fed with culture medium supplemented with 35 ng/ml M-CSF, and 60 ng/mL RANKL after five hours. Each value shown is determined in triplicate and repeated three times. Shown is the average.

### Bone resorption assays

Osteoclast precursors were plated on bone slices (NC1309388, Fisher Scientific) at a seeding density of 130,000 cells per well in culture medium supplemented with 35 ng/mL M-CSF and 60 ng/mL RANKL. Medium was replaced every 3–4 days. On Day 14, cells were lysed in 10% bleach washed 3 times with water. Pits on bone slices were stained with toluidine blue as previously described^73^. Resorption assays were performed in triplicate and repeated three times. Images were analyzed using ImageJ.

### TRAP staining

TRAP staining was performed as previously described^73^. Briefly, cells were fixed on cover glass with 10% neutral buffered formalin for 10 min and then washed 3 times with phosphate-buffered saline (PBS)^73^. Fixed cells were TRAP stained using the Acid Phosphatase, Leukocyte (TRAP) Kit (#387A-1KT, Sigma-Aldrich) and mounted to slides using Vectashield with DAPI (#H-1200, Vector Laboratories, Burlingame, CA)^73^. For osteoclastogenesis experiments, three cover glasses were used per experimental condition. For each cover glass, three fields were imaged using a 10× objective^73^. Images were digitally photographed using the Zeiss AxioVert A1 and Zen software package. Osteoclasts were defined as TRAP^+^ cells with 3 or more nuclei^73^. The number of mononuclear cells and osteoclasts, as well as osteoclast area and nuclei per osteoclast within each image were quantified using ImageJ or Photoshop software^73^. The percent multinucleated cells is the number of osteoclasts divided by the number of mononuclear cells within each field. The average number of nuclei per cell was evaluated by counting the number of DAPI stained nuclei per TRAP^+^ cell and dividing by the total number of cells. The number of TRAP^+^ cells with defined numbers of nuclei was also determined. Each experiment was repeated using cells derived from male or female mice independently, each repeated three times. Shown is the average.

### TUNEL and DAPI staining

TUNEL staining was performed using the Roche, In Situ Cell Death Detection Kit (Fluorescein) according to the manufacturer’s specifications (#11684795910, Sigma-Aldrich). DAPI staining was used as previously described to measure cell numbers^73^. To determine the percentage of TUNEL positive cells, the number of TUNEL-positive cells was divided by the number of DAPI positive cells in multiple regions.

### RNA extraction and qPCR

Total RNA was extracted from primary osteoclasts using TRIzol (#15596026, Invitrogen) and chloroform, and 1 μg of RNA was reverse transcribed using the iScript Reverse Transcription Supermix for RT-qPCR kit (#1708840, Bio-Rad, Hercules, CA). The resulting cDNAs were used to assay gene expression via real-time PCR using the following gene-specific primers: Chi3l3, (5′-GTACCCTGGGTCTCGAGGAA-3′, 5′-CCTTGGAATGTCTTTCTCCACAG-3′), Ctsk (5′- TCCGAAAAGAGCCTAGCGAA-3′, 5′- AGAGATTTCATCCACCTTGCTGT-3′), Hdac3 (5′-CCCGCATCGAGAATCAGAAC-3′, 5′-TCAAAGATTGTCTGGCGGATCT-3′), Pmepa1 (5′-ATGGAGATCACGGAGCTGGAGT-3′, 5′-GGCTGACAGCTTGTAGTGGC-3′), Rank (5′- TAAAGTCTGTGATGCAGGCAAG-3′, 5′-CCGTATCCTTGTTGAGCTGC-3′), Retnla (5′-AACTGCCTGTGCTTACTCGT-3′, 5′-CAAGAAGCAGGGTAAATGGGC-3′) Tubulin (5′-TGCTCATCAGCAAGATCAGAG-3′, 5′-GCATTATAGGGCTCCACCACAG-3′), and Ywhaz (5′-GCCCTAAATGGTCTGTCACC-3′, 5′-GCTTTGGGTGTGACTTAGCC-3′). Fold changes in gene expression for each sample were calculated using the 2^−ΔΔCq^ method relative to control following normalization of gene-specific C_q_ values to Tubulin or Ywhaz C_q_ values as noted^74^. Each value shown is determined in triplicate and repeated three times. Shown is the average.

### Western blotting

Western blotting was performed as previously described^41^. Briefly, cell lysates were collected in a buffered SDS solution (0.1% glycerol, 0.01% SDS, 0.1 m Tris, pH 6.8) on ice. Total protein concentrations were obtained with the Bio-Rad D_C_ assay (#5000112, Bio-Rad)^41^. Proteins (30 μg) were then resolved by SDS-PAGE and transferred to a polyvinylidene difluoride membrane^41^. Western blotting was performed with antibodies (1:2000 dilution) for anti-phospho-p65 NF-κB (#ab86299, Abcam, Cambridge, MA), anti-phospho-Mek1/2 (# 9121, Cell Signaling Technologies), anti-phospho-Erk1/2 (# 9101, Cell Signaling Technologies), anti-Mek1/2 (# 4694 Cells Signaling Technologies), anti-Erk1/2 (# 4370, Cell Signaling Technologies), anti-phospho-Ser423/Ser425-Smad3 (#ab52903, AbCam,), anti-Smad3 (#ab28379, AbCam), anti-Pmepa1 (#PA5-75980, Thermo Fisher Scientific, Waltham, MA), anti-Actin (#A2228, Sigma-Aldrich) and anti-Tubulin (E7, Developmental Studies Hybridoma Bank, Iowa City, IA) and corresponding secondary antibodies conjugated to horseradish peroxidase (HRP) (#7074S or #7076S, Cell Signaling Technology,)^41^. Antibody binding was detected with the Supersignal West Femto Chemiluminescent Substrate (#34096, Pierce Biotechnology, Rockford, IL)^41^. Each experiment was repeated using cells derived from male and female mice at least three times, and data from a representative experiment are shown.

### Unbiased high throughput RNA sequencing

Bone marrow macrophages were isolated from Hdac3 cKO_LysM_ male (n = 3) mice or their control littermates (n = 3) and cultured overnight with M-CSF. Non-adherent cells were then placed in osteoclastogenic conditions with RANKL and M-CSF as described above. On day 3, prior to cell fusion, cells were harvested for total RNA. Whole-transcriptome sequencing and bioinformatics analyses were performed as described previously^42^. The TruSeq RNA sample Prep Kit v2 (Illumina, San Diego, CA) was used to prepare RNA samples, and then samples were analyzed using the Illumina HiSeq 2000 with the TruSeq SBS sequencing kit version 3 and HCS v2.0.12 data collection software^42^. Following data collection, sequence data were processed using MAPRSeq (v.1.2.1) and the bioinformatics workflow (TopHat 2.0.6, HTSeq, and edgeR 2.6.2), where expression data were normalized using the reads per kilobase per million (RPKM) method^42^. Paired sample analysis was used to identify differentially expressed genes (n = 71) that had a two-fold or greater change with a false discover rate (FDR) less than 0.05^42^. One set of paired samples was excluded from the analysis due to insufficient reduction in Hdac3 RPKM values of the Hdac3 cKO_LysM_ sample as compared to control. Gene functional annotation analysis was performed using DAVID Bioinformatics Resources 6.7 (http://david.abcc.ncifcrf.gov).

### Bone histomorphometry

Femurs containing cortical bone defects (n = 5 WT, n = 6 Hdac3 cKO_LysM_) were decalcified, paraffin embedded and sectioned to a thickness of 7 µm. Sections were TRAP stained using the Acid Phosphatase, Leukocyte (TRAP) Kit (#387A-1KT, Sigma-Aldrich) or stained with Goldner’s trichrome stain (#1004850001, Sigma-Aldrich) according to the manufacture’s specifications^73^. The number of TRAP positive cells within the defect site or distal femur was determined, and standard bone histomorphometric measurements were collected as previously described^73^.

### Statistical analysis

Data obtained are the mean ± standard deviation. *p* values were determined with the Student's *t* test when only one experimental comparison was made. For assessment of significance with greater than two conditions, a one-way analysis of variance was performed. *p* < 0.05 was considered statistically significant unless otherwise noted^75^. Statistical analyses were performed using Graphpad Prism 7 software.

## Supplementary information

Supplementary Information 1.

## Abbreviations

IL: Interleukin
Hdac3: Histone deacetylase 3
cKO: Conditional knockout
Pmepa1: Prostrate transmembrane protein, androgen induced 1
Tgfβ: Transforming growth factor beta
IFNγ: Interferon gamma
LPS: l Lipopolysaccharides
TNFα: Tumor necrosis factor alpha
M-CSF: Macrophage-colony stimulating factor
RANKL: Receptor activator of nuclear factor kappa-B ligand
NFATc1: Nuclear factor of activated T cells
Arg1: Arginase 1
BMM: Bone marrow macrophage
SAHA: Suberoylanilide hydroxamic acid
BV/TV: Bone volume per total volume
TUNEL: Terminal deoxynucleotidyl transferase dUTP nick end labeling
DAVID: Database for Annotation, Visualization and Integrated Discovery
TRAP: Tartrate-resistant acid phosphatase
DAPI: 4′,6-Diamidino-2-phenylindole
Prss46: Serine protease 46
Gdf15: Growth and differentiation factor 15
PCR: Polymerase chain reaction
Smad: Sma and mothers against decapentaplegic
MAPK: Mitogen activated protein kinase
Ctsk: Cathepsin-K
CT: Computed tomography
RBC: Red blood cell
MEM: Minimal essential medium
FBS: Fetal bovine serum
PBS: Phosphate buffered saline
RNA: Ribonucleic acid
DNA: Deoxynucleic acid
Chi3l3: Chitinase-like 3
Retnla: Resistin like alpah
Ywhaz: Tyrosine 3-monooxygenase/tryptophan 5-monooxygenase activation protein, zeta polypeptide
SDS–PAGE: Sodium dodecyl sulfate–polyacrylamide gel electrophoresis
HRP: Horseradish peroxidase
RPKM: Reads per kilobase per million
FDR: False discovery rate
RANK: Receptor activator of NF-Κb
Ctsk: Cathepsin K
Mek1/2: Mitogen-activated protein kinase kinases 1/2
Erk1/2: Extracellular regulated kinases 1/2
