# Supplementary material for: Hdac3 deletion in myeloid progenitor cells enhances bone healing in females and limits osteoclast fusion via Pmepa1
Source: Sci Rep. 2020 Dec 11;10:21804. doi: 10.1038/s41598-020-78364-5 (PMC7733476; doi:10.1038/s41598-020-78364-5)
Supplement: Supplementary file 1 — Supplementary Information 1. [file 41598_2020_78364_MOESM1_ESM.pdf]

Hdac3 deletion in myeloid progenitor cells enhances bone healing in females and limits osteoclast fusion via Pmepa1

Supplemental Information

David H.H. Molstad[1], Elizabeth Zars[4], Andrew Norton[3], Kimberly C. Mansky[3], Jennifer J. Westendorf[4,5] and Elizabeth W. Bradley*[1,2]

From the [1]Department of Orthopedics, [2]Stem Cell Institute, and Department of Developmental and Surgical Sciences, University of Minnesota, Minneapolis, MN, [4]Departments of Orthopedic Surgery, [5]Biochemistry and Molecular Biology, Mayo Clinic, Rochester, MN

Running title: *Hdac3 controls osteoclastogenesis*

*To whom correspondence should be addressed: Elizabeth W. Bradley, 100 Church St. S.E., Minneapolis MN 55455, 612-301-2810, ebradle1@umn.edu

Keywords: Hdac3, osteoclast, Pmepa1, Tbfβ, Smad, bone, fracture

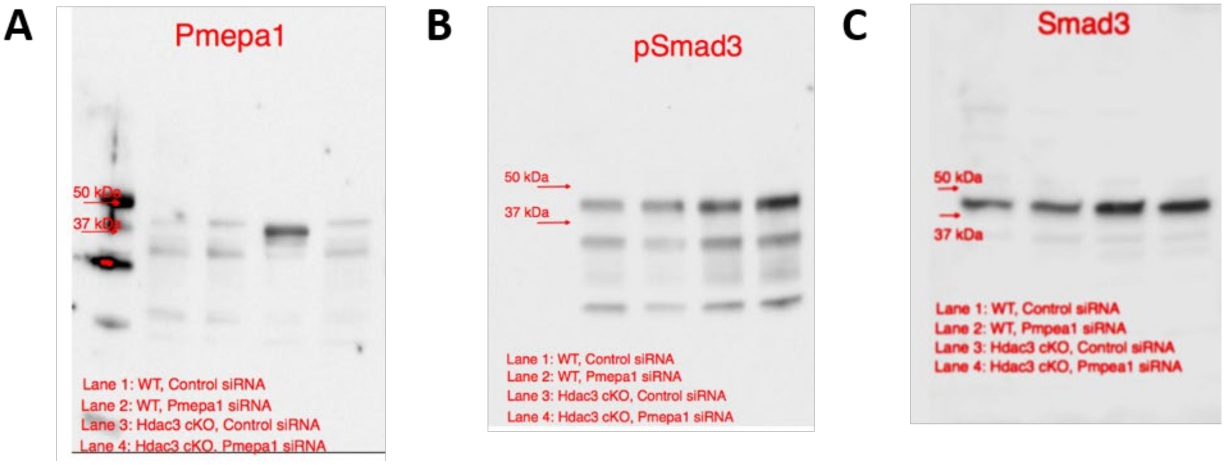

Supplementary Figure 1. This figure documents uncropped, annotated images of western blots shown in Figure 5. The membrane was sequentially probed using (A) anti-Pmepa1, (B) anti-phospho-Smad3 and (C) anti-Smad3 antibodies.

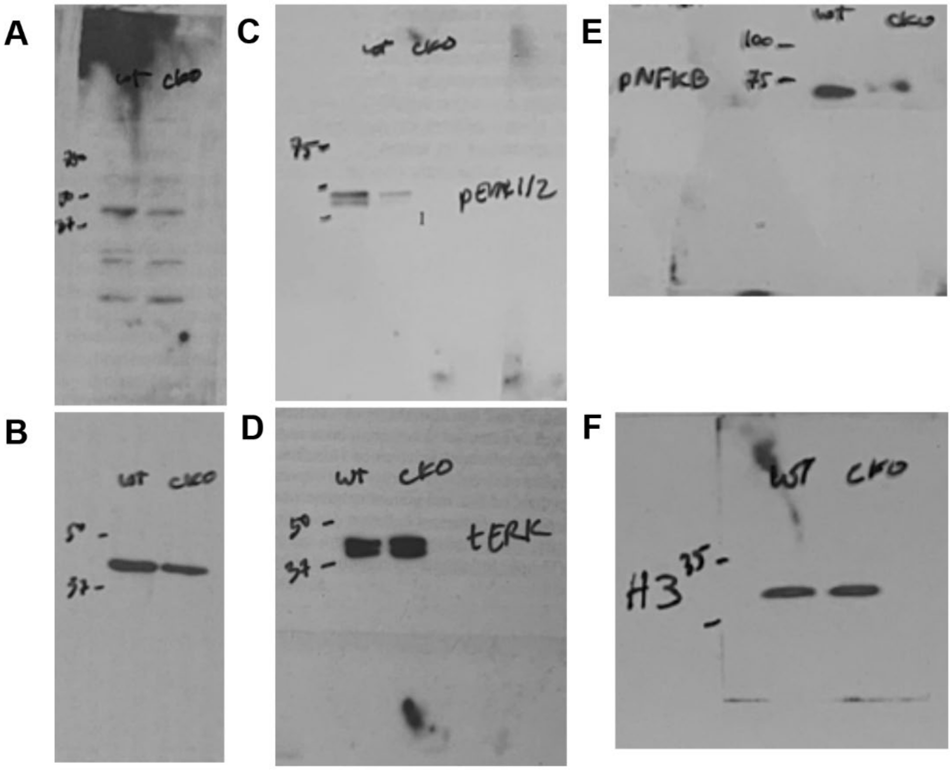

Supplementary Figure 2. This figure documents uncropped, annotated images of western blots shown in Figure 4. The membranes were sequentially probed using (A) anti-phosphor-Mek1/2 (A) and anti-Mek1/2 antibodies. Blots were also sequentially probed with anti-phospho-Erk1/2 (C) and anti-Erk1/2 or anti-phospho-p65 NF-κB (E) and anti-histone 3 (F) antibodies.
